# Should spring water cysts of the mediastinum require excisional resection? A case report

**DOI:** 10.1016/j.ijscr.2021.106293

**Published:** 2021-08-08

**Authors:** Soichiro Kiya, Kembu Nakamoto, Toshiyuki Fujii, Eriko Sakka, Yousuke Tsutsumi, Kazuya Yoshida

**Affiliations:** a Division of General Thoracic Surgery, Shunan Memorial Hospital, 1-10-1 Ikunoyaminami, Kudamatsu-shi, Yamaguchi 744-0033, Japan; b Division of Anesthesiology, Shunan Memorial Hospital, 1-10-1 Ikunoyaminami, Kudamatsu-shi, Yamaguchi 744-0033, Japan

**Keywords:** Mini-VATS, video-assisted thoracoscopy with a miniaturized endoscope, CT, chest computed tomography, MRI, magnetic resonance imaging, Spring water cysts, Mediastinal cysts, Fenestration, Mini-VATS, Case report

## Abstract

**Introduction and importance:**

Mediastinal cysts account for 20–32% of all mediastinal lesions. Complete surgical excision is the standard therapy for mediastinal cysts. Translucent cysts containing crystal-clear fluid are called “spring water cysts.” We experienced a case of mediastinal spring water cyst fenestrated under video-assisted thoracoscopy with a miniaturized endoscope (mini-VATS) as an alternative to excisional resection.

**Case presentation:**

A 49-year-old woman presented with back pain. Chest CT revealed a posterior mediastinal mass measuring 4.2 × 1.8 × 3.2 cm closed to the tenth thoracic vertebra. Chest MRI demonstrated hypo-intensity on T1-weighted images and hyper-intensity on T2-weighted images. It was estimated that the posterior mediastinal mass did not contain a tumor component. The tumor was growing and symptomatic; therefore, we performed surgical cyst fenestration without excision of the cyst under mini-VATS. The patient experienced complete relief of symptoms. Fluid accumulation in the cyst was not observed on CT images 12 months postoperatively.

**Clinical discussion:**

Kozu et al. reported that all 108 primary mediastinal cysts were resected completely and were recurrence-free after a mean follow-up of 41 ± 26 months. In the case of a functional hydrocele such as spring water cyst, we believe that even if fluid is produced, the thoracic pleura is capable of absorbing the fluid, and the cyst wall might not recur even if the wall is left in place. Fluid drainage through fenestration may prevent recurrent fluid collection.

**Conclusion:**

Fenestration of non-neoplastic mediastinal cysts under mini-VATS might be a less invasive radical procedure compared to complete resection.

## Highlights

• Translucent cysts containing crystal-clear fluid are called “spring water cysts.”
• Complete surgical excision is the standard therapy for mediastinal cysts.
• Fenestration of the cyst has lower risk of bleeding or injury to the adjacent normal structures than complete resection.
• Fenestration of non-neoplastic mediastinal cysts might be a less invasive radical procedure compared to complete resection.

## 1 Introduction

Mediastinal cysts account for 20–32% of all mediastinal lesions [1], including thymic cysts, bronchogenic cysts, mature cystic teratoma, pericardial cysts, and esophageal duplication cysts. The symptomatic rate for these cysts is approximately 20%. Asymptomatic mediastinal cysts are removed excisionally because of malignant transformation, cyst infection, progressive growth, or spontaneous rupture [2]. Complete surgical excision is the standard therapy for mediastinal cysts, with a postoperative complication rate of 6–12% [2], [3].

Translucent cysts containing crystal-clear fluid are called “spring water cysts.” We experienced a case of mediastinal spring water cyst fenestrated under video-assisted thoracoscopy with a miniaturized endoscope (mini-VATS) [4] as an alternative to excisional resection. We discuss the indication for surgery and the surgical procedure for non-neoplastic mediastinal cyst with crystal-clear fluid. This case report has been reported in line with the SCARE Criteria [5].

## 2 Case presentation

**Fig. 1:**
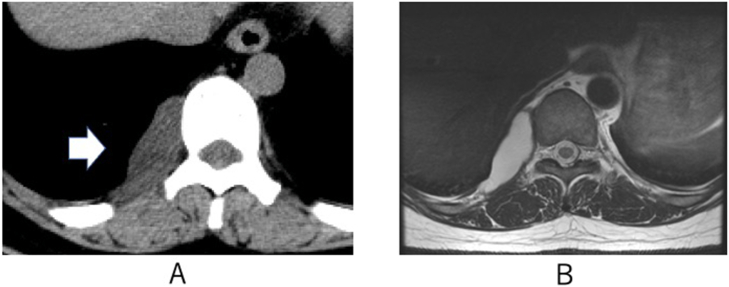
A: Chest computed tomography revealed a posterior mediastinal mass (arrow) measuring 4.2 × 1.8 × 3.2 cm close to the tenth thoracic vertebra. B: Chest magnetic resonance imaging revealed hypo-intensity on T1-weighted images and hyper-intensity on T2-weighted images.

A 49-year-old woman presented with back pain. She had no particular medical history, but she has suffered from lumbago since she was young. And she had no surgical or drug consumption history. There was no family history associated with this disease. Chest computed tomography (CT) revealed a posterior mediastinal mass measuring 4.2 × 1.8 × 3.2 cm closed to the tenth thoracic vertebra (Fig. 1A), while plain chest X-ray was normal. Chest magnetic resonance imaging (MRI) demonstrated hypo-intensity on T1-weighted images and hyper-intensity on T2-weighted images (Fig. 1B). It was estimated that the posterior mediastinal mass did not contain a tumor component. The tumor was growing and symptomatic; therefore, surgery was indicated.

**Fig. 2:**
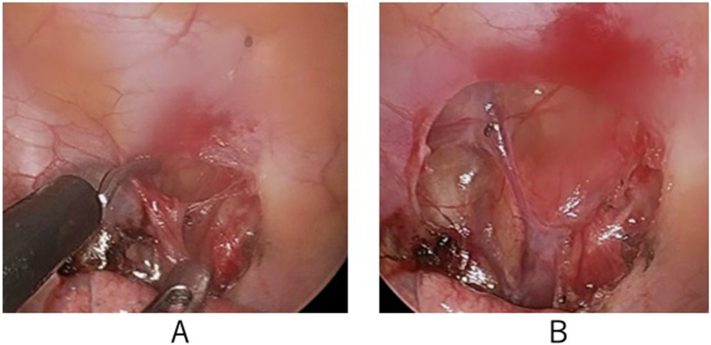
The intraoperative image of mini-VATS. A part of the mediastinal cyst wall has been removed with scissors (A). After fenestration of the cyst (B).

The patient underwent surgery under general anesthesia. The patient was placed in a lateral decubitus position and fixed with a fixing mattress (Magic Bed, Okada Medical Co. Ltd., Tokyo) on the operating table. An artificial pneumothorax for scope introduction was produced by needle thoracentesis under atmospheric pressure. Two ports were created at the seventh and eighth intercostal spaces; each skin incision was 2–5 mm long. A mini thoracoscope (2.9 mm IDEAL EYES, Stryker Co., Kalamazoo, MI) was used. The mediastinal semilucent cyst existed at the anterolateral portion of the tenth thoracic vertebra. The fluid in the cyst (10 mL) was absorbed using a 16G needle; rapid cytology of the fluid showed no malignancies. The fluid was watery and crystal-clear without cell particles. A coin-sized fenestration was created using a pair of scissors and electrocautery. There was a risk of hemorrhage at the anterior surface of the vertebral body when the whole cyst was dissected due to the exposure of blood vessels on the luminal surface of the cyst. Surgical cyst fenestration without excision of the cyst was performed under mini-VATS (Fig. 2A, B). At the end of the procedure, a double-lumen silicon catheter (18F Phicon Samp Catheter, Fuji Systems Co., Tokyo) was inserted into the thoracic cavity.

**Fig. 3:**
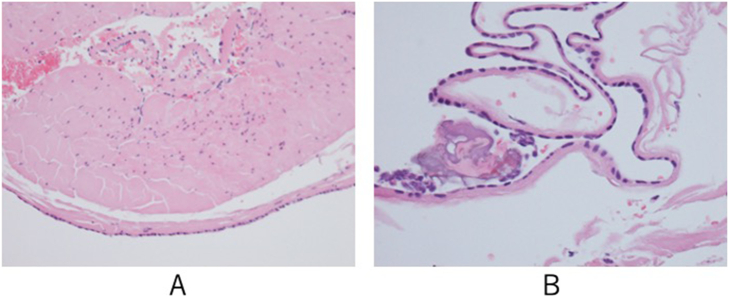
A single layer of mesothelial cell-covered cystic lesions with thicker collagenous tissue just below the mesothelial cells was observed, accompanied by the presence of vitrification. No evidence of malignancy was found. (A: ×200, B: ×400).

**Fig. 4:**
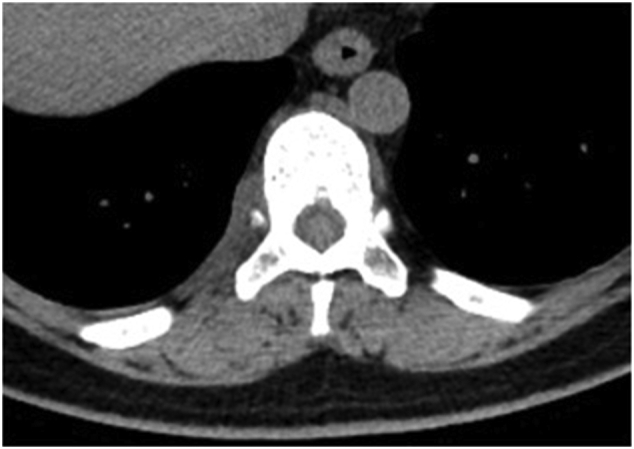
Chest computed tomography did not show fluid accumulation in the cyst 3 months after surgery.

The thoracic catheter was removed on postoperative day 1, and the patient was discharged on postoperative day 4. The postoperative course was uneventful. Histopathologic evaluation of the resected specimen revealed a mesothelial cyst (Fig. 3A, B), and the origin of the mediastinal cyst was pleura. The patient experienced complete relief of symptoms. Fluid accumulation in the cyst was not observed on CT images 12 months postoperatively (Fig. 4).

## 3 Discussion

### 3.1 Mediastinal spring water cysts

The appearance of the thin-walled, translucent cysts containing crystal-clear fluid gave rise to the name “spring water cysts.” Greenfield and colleagues introduced the new term into the medical literature when they encountered a cyst filled with “crystal-clear fluid of watery consistency” in 1943 [6]. Thin-walled solitary cysts with a low uniform density can be diagnosed as congenital thymic cysts according to their appearance on CT and MRI [7]. However, Margaret, et al. demonstrated a high proportion of agreement (78%) between the results of fine-needle aspiration biopsy from the mediastinum and subsequent histologic diagnoses for a wide variety of mediastinal lesions [8]. Therefore, the diagnosis of spring-water cysts can be confirmed by intraoperative cytology.

### 3.2 Mini-VATS

In this case, surgery was performed under general anesthesia as a complete resection with unilateral lung collapse was predicted. Fenestration may not require lung collapse during the procedure. Fenestration under mini-VATS is a relatively simple and less invasive procedure; local infiltration anesthesia or intercostal nerve block [9] may be applicable for tracheal intubation anesthesia. Moreover, mini-VATS is repeatedly applicable for the recurrence of non-neoplastic cysts because of its simplicity and lower invasiveness.

### 3.3 Complete resection vs. fenestration

Complete resection of the cyst has the risk of bleeding or injury to the adjacent normal structures. In this case, the cyst made contact with the vertebral column, and there was the risk of bleeding from the segmental artery and rupture of the vertebral foramen. Kozu et al. reported that all 108 primary mediastinal cysts were resected completely and were recurrence-free after a mean follow-up of 41 ± 26 months [3]. Moreover, Esme et al. reported that all 32 patients were asymptomatic and recurrence-free [2]. Spring water cyst could be categorized as a functional hydrocele. In the case of a functional hydrocele, we believe that even if fluid is produced, the thoracic pleura is capable of absorbing the fluid, and the cyst wall might not recur even if the wall is left in place. Fluid drainage through fenestration may prevent recurrent fluid collection.

## 4 Conclusion

Fenestration of non-neoplastic mediastinal cysts under mini-VATS might be a less invasive radical procedure compared to complete resection.

## Funding

No funding sources.

## Ethical approval

No approval is required for this case report.

## Consent

Written informed consent was obtained from the patient for publication of this case report and accompanying images. A copy of the written consent is available for review by the Editor-in-Chief of this journal on request.

## Registration of research studies

Not applicable.

## Provenance and peer review

Not commissioned, externally peer-reviewed.

## Guarantor

Soichiro Kiya, MD

Kembu Nakamoto, MD, PhD

## CRediT authorship contribution statement

Soichiro Kiya, MD: Surgeon performing the operation, writing of original article.

Kembu Nakamoto, MD, PhD: Revision and final approval of the manuscript.

Toshiyuki Fujii, MD, PhD: Revision of the manuscript.

Eriko Sakka, MD, PhD: Anesthesiologist of the operation, and revision of the manuscript.

Yousuke Tsutsumi, MD, PhD: Anesthesiologist of the operation, and revision of the manuscript.

Kazuya Yoshida, MD, PhD: Revision of the manuscript.

## Declaration of competing interest

None.
